# Simultaneous confidence interval construction for many-to-one of proportion ratios of bilateral correlated data

**DOI:** 10.1371/journal.pone.0311850

**Published:** 2024-10-15

**Authors:** Zhaoqi Zhang, Chang-Xing Ma

**Affiliations:** Department of Biostatistics, University at Buffalo, Buffalo, New York, United States of America; Indian Institute of Management Kozhikode, INDIA

## Abstract

In ophthalmology and otolaryngology, data collected from paired body parts are typically reformatted into categorical bilateral data structures for subsequent research. This article applies Donner’s equal correlation coefficient model and obtains nine simultaneous confidence intervals (SCI) of proportion ratios under three asymptotic statistical methods and three ways of multiplicity adjustment. The empirical coverage probability and mean interval width are evaluated through Monte Carlo simulations. A real example is used to demonstrate the proposed methods.

## Introduction

In the fields of ophthalmology and otolaryngology, data collected from paired body parts are typically reformatted into categorical bilateral data structures for subsequent research. For instance, visual acuity and intraocular pressure are typically measured from both eyes of a patient. The outcome would be bilateral responses, unilateral responses or no response. In this situation, the outcomes from both eyes of each patient tend to be highly correlated. Failing to consider intraclass correlation during data analysis can result in inaccurate findings.

Multiple methodologies and approaches have been developed over the last few decades to address the issue of correlated data [1]. Rosner [2] proposed a constant R model, which assumes the probability of a response on one side of the body part, given a response on the opposite side, is proportional to the prevalence rate of the corresponding group in the research study. Tang et al. [3] examined the performance of various methods for assessing the equality of proportions, focusing on asymptotic and approximate unconditional approaches. Their results indicated that the approximate unconditional score test performs well in general scenarios. Additionally, Tang [4] and Xue [5, 6] developed multiple test statistics to assess proportion differences and proportion ratios, as well as to construct confidence intervals for these parameters. Moreover, Wang and Shan [7] developed twelve exact methods for constructing CIs for relative risk and odds ratio, which resulted in shorter interval lengths.

Dallal’s study [8] identified a limitation inherent in the constant R model, pointing out that it doesn’t fit well when a characteristic displays high variability across different groups and often occurs bilaterally. He addressed the issue by proposing that the model’s assumption of constant conditional probability is not proportional to the prevalence rate. Subsequently, Donner [9] proposed an equal correlation coefficient model, also known as the *ρ* model, predicated on the assumption of a common correlation coefficient among paired body ports within each group.

For the *ρ* model, numerous studies have been proposed. For instance, Ma and Liu [10] developed a common test to examine the equality of proportion among multiple groups, utilizing three statistical methods. They recommended the score test as the most reliable approach. Beyond statistical tests, CI approaches offer a more straightforward alternative, providing a range of values within which the true parameter value is likely to fall. Pei et al. [11] introduced five asymptotic CIs approaches for measuring the proportion differences between two groups, recommending the Wald-type CI with an assumption of dependence as the most robust option. Afterward, Li and Ma [12], Shen [13] and Zhuang [14] developed a common test for odds ratios; multiple CIs approaches to analyze odds ratios, proportion differences, and proportion ratio in a two-group scenario.

In random clinical trials, there is a trend to include multiple treatment groups alongside a control group. This design allows researchers to evaluate the collective effects of various treatments simultaneously or to evaluate a new therapy against several established alternatives. For instance, a multiple-dose study may be required to determine the bioavailability. In this context, the use of simultaneous confidence intervals (SCIs) provides a methodology for many-to-one comparisons. Yang [15] and Peng [16] introduce asymptotic SCIs for the proportion differences and odds ratio based on the constant R model. Later, Yang [17] developed asymptotic SCIs for the proportion difference based on the *ρ* model.

In addition to proportion differences and odds ratios, proportion ratios are also crucial for assessing the relative strength of associations between groups, especially when the corresponding proportions are small. To address the need for further research, this article extends the discussion to asymptotic SCIs for proportion ratios in settings with g (where g≥ 2) groups based on the *ρ* model.

The rest of the article is structured in the following manner. The Methods section introduces three methods for constructing SCIs (Wald-type SCI, profile likelihood SCI, and asymptotic score SCI) and the multiplicity adjustment methods. In the Simulation studies section, simulation experiments are conducted to evaluate the performance of the proposed methods, with comparisons made based on the empirical coverage probability and the mean interval width. The Real case example section uses a real data example to illustrate the methodology proposed in this article. Finally, the last section offers a discussion of the results.

## Methods

### Data structure

Suppose the object of this study is to evaluate the effectiveness and safety of multiple new treatments for eye disease against a standard treatment or placebo. Let *m*_*li*_ be the number of patients in the *i*^*th*^ group (*i* = 1,2,⋯, *g*) with l responses (l = 0,1,2), and *m*_*i*_ be the total number of patients in the *i*^*th*^ group, which is assumed to be fixed. Let *S*_*l*_ be the total number of patients with l responses. Let N be the total number of patients in the research study. The data structure is summarized in Table 1.

**Table 1:** Data structure for the correlated bilateral data.

Number of responses(l)	Group				Total
	1	2	⋯	g	
0	*m*_01_	*m*_02_	⋯	*m*_0*g*_	*S*_0_
1	*m*_11_	*m*_12_	⋯	*m*_1*g*_	*S*_1_
2	*m*_21_	*m*_22_	⋯	*m*_2*g*_	*S*_2_
**Total**	*m*_1_	*m*_2_	⋯	*m*_*q*_	N

According to the constant correlation coefficient model proposed by Donner [9], the disease rates are assumed to be the same in the same group.

Let *Z*_*ijk*_ be the dummy variable of the response of the *k*^*th*^ body part (eg. eye) (k = 1,2) of the *j*^*th*^ individual in the *i*^*th*^ group (i = 1,2,⋯, *g*). Let *ρ* be the common correlation coefficient.

$$\begin{array}{c} Z_{ijk}=\{\begin{array}{cc} 1, & \text{event(disease)}\;\text{occur}.\\ 0, & \text{otherwise}. \end{array} \end{array}$$

$$\begin{array}{c} \text{Pr}(Z_{ijk}=1)=\pi_{i};i=1,\ldots ,g;j=1,\ldots ,m_{i};0\leq \pi_{i}\leq 1\\ \text{Corr}(Z_{ijk},Z_{ij(3-k)})=\rho ,0\leq \rho \leq 1 \end{array}$$

Each group of the data follows a multinomial distribution. The probability density function of observation frequencies *m*_*i*_ = (*m*_0*i*_, *m*_1*i*_, *m*_2*i*_) is defined as follows,

$$\begin{array}{c} f(m_{0i},m_{1i},m_{2i})=\frac{m_{i}!}{m_{0i}!m_{1i}!m_{2i}!}p_{0i}^{m_{0i}}p_{1i}^{m_{1}}p_{2i}^{m_{2i}} \end{array}$$

Let *p*_*li*_ be the corresponding probability for an individual in the *i*^*th*^ group has exactly l events (*l* = 0, 1, 2, i = 1, 2,⋯, *g*) happened,

$$\begin{array}{c} \left\{ \begin{array}{cc} & p_{0i}=\left(1-\pi_{i}\right)\left(\rho \pi_{i}-\pi_{i}+1\right)\\ & p_{1i}=2\pi_{i}\left(1-\rho \right)\left(1-\pi_{i}\right),\\ & p_{2i}=\pi_{i}^{2}+\rho \pi_{i}\left(1-\pi_{i}\right), \end{array}\right. \end{array}$$ (1)

and *p*_0*i*_ + *p*_1*i*_ + *p*_2*i*_ = 1 for any fixed i.

Without loss of generality, let the 1^*th*^ group be the control, denotes the ratio of proportions between any treatment groups and control group by *δ*_*i*_ = *π*_*i*_/*π*_1_(*i* = 2, …, *g*), and for all pairwise comparisons by *δ*_*ij*_ = *π*_*i*_/*π*_*j*_ (i ≠ j).

The corresponding log-likelihood function can be expressed as:

$$\begin{array}{c} \begin{array}{cc} l_{1}\left(\pi_{1},\cdots ,\pi_{g};\rho \right)= & \sum_{i=1}^{g}[m_{2i}\;\text{log}({\pi_{i}}^{2}-\pi_{i}\;\rho \;(\pi_{i}-1))\\ + & m_{1i}\;\text{log}(2\;\pi_{i}\;(\pi_{i}-1)\;(\rho -1))\\ + & m_{0i}\;\text{log}(-(\pi_{i}-1)\;(\pi_{i}\;\rho -\pi_{i}+1))]+\text{log}\;C \end{array} \end{array}$$ (2)

where $\text{C}=\prod_{i=1}^{g}\left(\frac{m_{i}!}{m_{0i}!m_{1i}!m_{2i}!}\right)$ is a constant.

Substituting *π*_*i*_ = *π*_1_ *δ*_*i*_(*i* = 2, ⋯, *g*) into *l*_1_, the log-likelihood function can be written as

$$\begin{array}{c} \begin{array}{cc} l_{2}\left(\delta_{i},\pi_{1},\cdots ,\pi_{g};\rho \right) & =\sum_{j=1,j\neq i}^{g}[m_{2j}\;\text{log}({\pi_{j}}^{2}-\pi_{j}\;\rho \;(\pi_{j}-1))\\ & +m_{1j}\;\text{log}(2\;\pi_{j}\;(\pi_{j}-1)\;(\rho -1))\\ & +m_{0j}\;\text{log}(-(\pi_{j}-1)\;(\pi_{j}\;\rho -\pi_{j}+1))]\\ & +m_{2i}\;\text{log}({\left(\pi_{1}\delta_{i}\right)}^{2}-\pi_{1}\delta_{i}\;\rho \;(\pi_{1}\delta_{i}-1))\\ & +m_{1i}\;\text{log}(2\;\pi_{1}\delta_{i}\;(\pi_{1}\delta_{i}-1)\;(\rho -1))\\ & +m_{0i}\;\text{log}(-(\pi_{1}\delta_{i}-1)\;(\pi_{1}\delta_{i}\;\rho -\pi_{1}\delta_{i}+1)) \end{array} \end{array}$$ (3)

where *δ*_*i*_(*i* = 2, ⋯, *g*) is the parameter of interest, *π*_*j*_(*j* ≠ *i*) and *ρ* are nuisance parameters.

### Multiplicity adjustment

For the construction of SCIs, without considering multiplicity adjustment, the type I error rate will increase, as well as false-positive errors. Based on the data structure mentioned previously, there are *g* groups in total, one control group and *g*-1 treatment groups. The main purpose of measuring the effectiveness of each treatment is to compare the proportion ratio between the treatment and the control group. One method used in this paper to control multiplicity adjustment is the Bonferroni correction, the quantile *c* = *z*_1−*α*/2(*g*−1)_, where *z* denotes the standard normal distribution.

Another method used in this paper is the Sidak correction [18], which represents a modification of the Bonferroni correction. This correction involves the quantile $c=z_{1-{\left(1-\alpha \right)}^{1/g}}$, where *z* denotes the standard normal distribution.

The other method used in this paper is based on Dunnett test. Piergorsch [19] proposed a general method for constructing SCIs for pairwise proportion differences. let *o*_*i*_ = log *π*_*i*_ for the = *i*^*th*^ group, *o*_2_ − *o*_1_,…,*o*_*g*_ − *o*_1_ are simultaneously compared, the SCI of proportion ratio *δ*_*i*_ = *π*_*i*_/*π*_1_ is obtained by exponentiating the previous outcome. The critical value $\text{c}={\left|z\right|}_{g-1;R}^{\alpha }$ equals to 1-*α*/2 quantile of *g* − 1 variate normal distribution with mean equal to zero and correlation matrix R = {*ρ*_*ij*_}, *ρ*_*ij*_ = *ω*_*i*_ *ω*_*j*_, and

$$\omega_{i}={\left[1+\frac{m_{1}}{m_{i}}\frac{\hat{\pi_{i}}(1-\hat{\pi_{i}})}{\hat{\pi_{i}}(1-\hat{\pi_{i}})}\right]}^{-1/2}$$

### Wald-type interval

Ma and Liu [10] proposed a third-order polynomial and Fisher scoring method to derive the maximum likelihood estimator (MLE) of (*π*_1_, …, *π*_*g*_; *ρ*). After that, we can derive the MLE of proportion ratio (***δ***) through a simple linear transformation form log(*π*_*i*_) based on the invariant property of MLE. Let ***β*** = (log(*π*_1_), …, log(*π*_*g*_), log(*ρ*)) and the corresponding MLE of ***β*** is $\hat{\beta }=(log(\hat{\pi_{1}}),\ldots ,log(\hat{\pi_{g}}),log(\hat{\rho }))$, then the MLE of log(*δ*_*i*_) is $log(\hat{\delta_{i}})=K_{i}\hat{\beta^{T}}$, where

$$K_{\left(g-1)\times (g+1\right)}=\left[\begin{array}{ccccccc} -1 & 1 & 0 & \ldots & 0 & 0 & 0\\ -1 & 0 & 1 & \ldots & 0 & 0 & 0\\ \vdots & \vdots & \vdots & \ddots & \vdots & \vdots & \vdots \\ -1 & 0 & 0 & \ldots & 1 & 0 & 0\\ -1 & 0 & 0 & \ldots & 0 & 1 & 0 \end{array}\right]$$

*K*_*i*_ means the *i*^*th*^ row of the matrix *K*. The standard error of log(*π*_*i*_),i = 1,2,…,g, can be derived from $\hat{\pi_{i}}$ using delta method.

Let *γ* = (*π*_1_, ⋯, *π*_*g*_, *ρ*), under regularity conditions, the asymptotic distribution of *γ* is given by

$$\sqrt{n}\left(\hat{\gamma }-\gamma \right)\overset{\text{d}}{\to }N\left(0,I^{-1}\right)$$

where I is the Fisher information matrix of *γ*. See S1 Appendix for detail.

By delta method,

$$\sqrt{n}\left(\hat{\beta }-\beta \right)\overset{\text{d}}{\to }N\left(0,gI^{-1}g^{T}\right)$$

where $\text{g}=\text{Diag}(\frac{1}{\pi_{1}},\ldots ,\frac{1}{\pi_{g}},\frac{1}{\rho }$). The 100(1-*α*)% SCI for log(*δ*_*i*_) is given by

$$K_{i}{\hat{\beta }}^{T}\pm c\sqrt{K_{i}\left[g\hat{I^{-1}}g^{T}\right]K_{i}^{T}}$$

The 100(1-*α*)% SCI for *δ*_*i*_ is given by

$${\text{exp}}^{\left(K_{i}{\hat{\beta }}^{T}\pm c\sqrt{K_{i}\left[g\hat{I^{-1}}g^{T}\right]K_{i}^{T}}\right)}$$

where c is the critical value. If Bonferroni method is used, *c* = *z*_1−*α*/2(*g*−1)_, where *z* denotes the standard normal distribution. If Sidak method is used, *α*′ = 1 − (1 − *α*)^(1/*g*)^, *c* = *z*_1−*α*′/2_, where *z* denotes the standard normal distribution. If Dunnett method is used, c = ${\left|z\right|}_{g-1;R}^{\alpha }$, where |*z*| denotes 1-*α*/2 quantile of *g* − 1 variate normal distribution described in the previous section.

### Profile likelihood confidence interval

The asymptotic profile likelihood SCI for each proportion ratio (*δ*_*i*_, *i* = 2, …, *g*), can be constructed by inverting the likelihood ratio test of hypothesis *H*_0_ : *δ*_*i*_ = *δ*_0_ vs. *H*_*α*_ : *δ*_*i*_ ≠ *δ*_0_, *i* = 2, …, *g*. To simplify the explanation, we first set *i* = 2, when constructing the SCI, we start by calculating the CI for the ratio (*δ*_2_ = *π*_2_/*π*_1_) between the 2^*nd*^ group and the control group.

Let (${\tilde{\delta }}_{2},{\tilde{\pi }}_{1},{\tilde{\pi }}_{3},...,{\tilde{\pi }}_{g},\hat{\rho }$) denotes the constrained MLEs of (*δ*_2_, *π*_1_, *π*_3_, …, *π*_*g*_, *ρ*) under the null hypothesis, and (${\hat{\delta }}_{2},{\hat{\pi }}_{1},{\hat{\pi }}_{3},\ldots ,{\hat{\pi }}_{g},\hat{\rho }$) denotes the unconstrained MLEs under the alternative hypothesis. The likelihood ratio test statistic is given by

$$T_{L}=2[l_{2}\left({\hat{\delta }}_{2},{\hat{\pi }}_{1},{\hat{\pi }}_{3},\ldots ,{\hat{\pi }}_{g},\hat{\rho }\right)-l_{2}\left({\tilde{\delta }}_{2},{\tilde{\pi }}_{1},{\tilde{\pi }}_{3},\ldots ,{\tilde{\pi }}_{g},\tilde{\rho }\right)].$$

By solving the equations:

$$\begin{array}{ll} {\left.\frac{\partial l_{2}}{\partial \pi_{i}}\right|}_{\delta_{2}=\delta_{0}} & =0,(i=1,3,\ldots ,g)\\ {\left.\frac{\partial l_{2}}{\partial \rho }\right|}_{\delta_{2}=\delta_{0}} & =0 \end{array}$$

(${\tilde{\pi }}_{1},{\tilde{\pi }}_{3},\ldots ,{\tilde{\pi }}_{g},\tilde{\rho }$) can be computed given the constraints that *δ*_2_ = *δ*_0_. Since there is no closed-form solution, we adopt the Fisher-scoring method [14].

$$[\begin{array}{c} \pi_{1}^{\left(t+1\right)}\\ \pi_{3}^{\left(t+1\right)}\\ \vdots \\ \pi_{g}^{\left(t+1\right)}\\ \rho^{\left(t+1\right)} \end{array}]=[\begin{array}{c} \pi_{1}^{\left(t\right)}\\ \pi_{3}^{\left(t\right)}\\ \vdots \\ \pi_{g}^{\left(t\right)}\\ \rho^{\left(t\right)} \end{array}]+{I^{-1}(\pi_{1}^{\left(t\right)},\pi_{3}^{\left(t\right)},\ldots ,\pi_{g}^{\left(t\right)},\rho^{\left(t\right)})[\begin{array}{c} \frac{\partial l_{2}}{\partial \pi_{1}}\\ \frac{\partial l_{2}}{\partial \pi_{3}}\\ \vdots \\ \frac{\partial l_{2}}{\partial \pi_{g}}\\ \frac{\partial l_{2}}{\partial \rho } \end{array}]|}_{\left(\pi_{i}=\pi_{i}^{\left(t\right)},\rho =\rho^{\left(t\right)}\right)}$$

where $I(\pi_{1}^{\left(t\right)},\pi_{3}^{\left(t\right)},\ldots ,\pi_{g}^{\left(t\right)},\rho^{\left(t\right)})$ is the *g* × *g* Fisher information matrix estimated under the condition of (*π*_1_, *π*_3_, …, *π*_*g*_, *ρ*) = ($\pi_{1}^{\left(t\right)},\pi_{3}^{\left(t\right)},...,\pi_{g}^{\left(t\right)},\rho^{\left(t\right)}$). See S1 Appendix for detail. The test statistic follows a chi-square distribution with one degree of freedom, the 100(1 − *α*)% profile likelihood SCI for proportion ratio(*δ*_*i*_) satisfies

$$2(l_{2}\left({\hat{\delta }}_{2},{\hat{\pi }}_{1},{\hat{\pi }}_{3},\ldots ,{\hat{\pi }}_{g},\hat{\rho }\right)-l_{2}\left(\delta_{0},{\tilde{\pi }}_{1},{\tilde{\pi }}_{3},\ldots ,{\tilde{\pi }}_{g},\tilde{\rho }\right))\leq \chi_{1-\alpha /2(g-1)}^{2},$$

where $\chi_{1-\alpha /(g-1)}^{2}$ is the 1 − *α*/2(*g* − 1) quantile of the chi-square distribution with one degree of freedom with Bonferroni multiplicity adjustment, since $\chi_{1-\alpha /(g-1)}^{2}={\left(z_{1-\alpha /2(g-1)}\right)}^{2}$. Similarly, Sidak method can be applied bu substituting the critical value $\chi_{1-\alpha /(g-1)}^{2}$ with *χ*_1−*α*′_, where *α*′ = 1 − (1 − *α*)^(1/*g*)^. Dunnet’s multiplicity adjustment method can be applied by substituting the critical value *z*_1−*α*/2(*g*−1)_ with ${\left|z\right|}_{g-1;R}^{\alpha }$.

To compute the confidence interval and establish the lower and upper bounds, the following algorithm could be utilized to identify two roots:

To obtain the larger root, which is the upper bound of the CI.

1. Initiate parameters: Calculate the unconstrained MLEs (${\hat{\delta }}_{2},{\hat{\pi }}_{1},{\hat{\pi }}_{3},\ldots ,{\hat{\pi }}_{g},\hat{\rho }$) as the initial value $\left(\delta_{2}^{\left(0\right)},\pi_{1}^{\left(0\right)},\pi_{3}^{\left(0\right)},\ldots ,\pi_{g}^{\left(0\right)},\rho^{\left(0\right)})\right)$. Set initial value *flag* = 1, and *stepsize* = 0.1.
2. Update estimates: Updating ${\hat{\delta_{2}}}^{\left(1\right)}=\delta_{2}^{\left(0\right)}+flag\times stepsize$. And compute the constrained MLEs $\left({\tilde{\pi_{1}}}^{\left(1\right)},{\tilde{\pi_{3}}}^{\left(1\right)},\ldots ,{\tilde{\pi_{g}}}^{\left(1\right)},{\tilde{\rho }}^{\left(1\right)}\right)$ with $\delta_{2}={\hat{\delta_{2}}}^{\left(1\right)}$.
3. Evaluate the test statistics:
  If $2\times flag\times [l_{2}\left({\hat{\delta }}_{2},{\hat{\pi }}_{1},{\hat{\pi }}_{3},\ldots ,{\hat{\pi }}_{g},\hat{\rho }\right)-l_{2}\left({\hat{\delta }}_{2}^{\left(1\right)},{\hat{\pi }}_{1}^{\left(1\right)},{\hat{\pi }}_{3}^{\left(1\right)},\ldots ,{\hat{\pi }}_{g}^{\left(1\right)},{\hat{\rho }}^{\left(1\right)}\right)]<flag\times \chi_{1-\alpha /(g-1)}^{2}$, return to step 2. Updating ${\hat{\delta_{2}}}^{\left(t+1\right)}={\hat{\delta_{2}}}^{\left(t\right)}+\text{flag}\times stepsize$. Otherwise decrease the *stepsize* to 0.1 × *stepsize* and set *flag* = −*flag*.
4. Check for Convergence: If the *stepsize* is small enough (eg. 10^−5^), indicating convergence, return ${\hat{\delta }}_{2}^{\left(t+1\right)}$ as the upper bound of CI and stop iterating.

To obtain the smaller root, which is the lower bound of the CI, repeat steps 1–4, with the initial value *flag* = -1.

### Asymptotic score confidence interval

Under hypothesis *H*_0_ : *δ*_*i*_ = *δ*_0_ vs. *H*_*α*_ : *δ*_*i*_ ≠ *δ*_0_, *i* = 2, …, *g*, the asymptotic score test statistic can be derived as

$$T_{S}^{2}=UI^{-1}U^{T}{|}_{H_{0}}.$$

To simplify the explanation, we continue use the setting *i* = 2, where the score vector:

$$U=(\frac{\partial l_{2}}{\partial \delta_{2}},\frac{\partial l_{2}}{\partial \pi_{1}},\frac{\partial l_{2}}{\partial \pi_{3}},\ldots ,\frac{\partial l_{2}}{\partial \pi_{g}},\frac{\partial l_{2}}{\partial \rho }),$$

and I is the Fisher information matrix for (*δ*_2_, *π*_1_, *π*_3_, …, *π*_*g*_, *ρ*)^*T*^. Since *δ*_2_ is the parameter of interest, and *π*_*i*_, *ρ* are nuisance parameters, the score test statistic can be rewritten as:

$$T_{S}^{2}={{\left(\frac{\partial l_{2}}{\partial \delta_{2}}\right)}^{2}I^{-1}\left(1,1\right)|}_{\delta_{i}=\delta_{0}},$$

*I*(1, 1) denotes the (1, 1)^*th*^ element of *I*. See S1 Appendix for detail. *T*_*S*_ is asymptotically distributed as a chi-square distribution with one degree of freedom. The 100(1-*α*)% SCI for proportion ratio (*δ*_*i*_) satisfies

$$T_{S}^{2}={{\left(\frac{\partial l_{2}}{\partial \delta_{2}}\right)}^{2}I^{-1}\left(1,1\right)|}_{\delta_{i}=\delta_{0}}\leq \chi_{1-\alpha /2(g-1)}^{2}.$$

In a similar manner, the CI of the ratio needs to be determined using the iterative method described in the previous section. For each iteration, score test statistic is updated with a new Fisher information matrix, $I=I({\hat{\delta_{2}}}^{\left(t\right)},{\hat{\pi_{1}}}^{\left(t\right)},{\hat{\pi_{3}}}^{\left(t\right)},\ldots ,{\hat{\pi_{g}}}^{\left(t\right)},{\hat{\rho }}^{\left(t\right)})$. To address multiplicity, we use Bonferroni and Dunnett methods, as outlined in the previous section.

## Simulation studies

The performance of the proposed methods for constructing confidence intervals is evaluated through Monte Carlo simulation studies, utilizing empirical coverage probability (ECP) and mean interval width (MIW) as evaluation metrics. Both balanced (all *m*_*i*_’s are equal) and unbalanced (*m*_*i*_’s are different) designs are considered. The study’s parameter configurations are presented in Table 2.

**Table 2:** Parameter configuration setting for a simulation study.

group	*ρ*	*π*_1_, *π*_2_, ⋯, *π*_*g*_	*m*_1_, *m*_2_, ⋯, *m*_*g*_
*g* = 3	0.3,0.5,0.7	a. (0.4,0.4,0.4)	I. (50,50,50)
		b. (0.25,0.375,0.5)	II. (30,50,80)
			III. (30,100,500)
*g* = 4		a. (0.4,0.4,0.4,0.4);	I. (50,50,50,50)
		b. (0.25,0.375,0.425,0.5)	II. (30,50,80,100)
			III. (30,50,100,500)
*g* = 5		a. (0.4,0.4,0.4,0.4,0.4)	I. (50,50,50,50,50)
		b. (0.25,0.375,0.425,0.5,0.54)	II. (30,45,60,75,90)
			III. (30,50,100,200,500)

The study generate 10,000 replications for each configuration setting and construct 95% confidence intervals. All tests are conducted at a 5% significance level. The ECP is the proportion of sample replicates generated under the null hypothesis (*H*_0_) where the true value of the ratio (*δ*) is contained within the constructed CI. The MIW is the average of all widths of the SCIs across all replicates. A CI method is considered conservative when the ECP is significantly greater than the pre-specified nominal level of 1-*α*, liberal when the ECP is significantly less than 1-*α*, and recommended when the ECP is approximately at 1-*α*.

Tables 3–5 provide the ECPs and MIWs for group *g* = 3, 4, and 5, respectively. The ECP of the Score-Dunnett method closely aligns with the pre-specified nominal level (0.95) across all configurations. Although the Wald-Sidak method demonstrates a competitive advantage across all configurations, the Sidak adjustment method does not perform well when combined with the profile likelihood and the score method. The Wald-Dunnett, Profile-Bonferroni, Profile-Dunnett, and Score-Bonferroni methods have ECPs approximately equal to 0.95 in most configurations. The Wald-Bonferroni method is conservative since its ECP is greater than 0.95 across most configurations. Similarly, the Profile-Sidak method and Score-Sidak method also show conservative behavior, with their ECPs consistently above 0.95 in various settings. The Profile-Dunnett method reveals a tendency toward liberal behavior in some configurations. Additionally, in balanced cases, ECP is typically closer to the nominal level, with a shorter MIW. In general, the Dunnet method achieves better performance than the Bonferroni method in multiplicity adjustment. Therefore, SCI produced from the Score method with Dunnet multiplicity adjustment is strongly recommended.

**Table 3:** The empirical coverage probability (ECP) and the mean interval width (MIW) of 95% CI for proportion ratio (*g* = 3).

*ρ*,***π***,m			Wald-Bonferroni		Wald-Sidak		Wald-Dunnet	
			ECP	MIW	ECP	MIW	ECP	MIW
0.3	a	I	0.9569	0.9418	0.9419	0.8882	0.9524	0.9281
		II	0.9583	1.0908	0.9453	1.0276	0.9525	1.0592
	b	I	0.9628	2.0614	0.9477	1.9439	0.9576	2.0119
		II	0.9618	2.4924	0.9513	2.3563	0.9548	2.3979
		III	0.9667	2.3562	0.9538	2.2607	0.9538	2.2185
0.5	a	I	0.9570	1.0242	0.9416	0.9653	0.9535	1.0090
		II	0.9595	1.1948	0.9472	1.1256	0.9531	1.1603
	b	I	0.9599	2.2388	0.9451	2.1103	0.9533	2.1840
		II	0.9632	2.6767	0.9520	2.5401	0.9552	2.5843
		III	0.9651	2.5449	0.9549	2.4221	0.9520	2.4009
0.7	a	I	0.9605	1.1034	0.9446	1.0394	0.9569	1.0867
		II	0.9612	1.2941	0.9469	1.2182	0.9552	1.2558
	b	I	0.9617	2.4003	0.9468	2.2706	0.9549	2.3487
		II	0.9654	2.8283	0.9541	2.6911	0.9581	2.7390
		III	0.9664	2.6919	0.9549	2.5712	0.9533	2.5323
*ρ*,***π***,m			Profile-Bonferroni		Profile-Sidak		Profile-Dunnet	
			ECP	MIW	ECP	MIW	ECP	MIW
0.3	a	I	0.9499	0.9608	0.9641	1.0312	0.9465	0.9462
		II	0.9516	1.1539	0.9681	1.2474	0.9447	1.1169
	b	I	0.9552	2.1503	0.9694	2.3126	0.9493	2.0944
		II	0.9595	2.6749	0.9715	2.8638	0.9485	2.5696
		III	0.9621	2.5320	0.9698	2.7370	0.9492	2.3723
0.5	a	I	0.9488	1.0466	0.9630	1.1246	0.9445	1.0304
		II	0.9497	1.2723	0.9656	1.3768	0.9423	1.2297
	b	I	0.9510	2.3327	0.9659	2.5038	0.9439	2.2735
		II	0.9570	2.8481	0.9697	3.0402	0.9468	2.7478
		III	0.9570	2.7368	0.9677	2.9060	0.9410	2.5651
0.7	a	I	0.9500	1.1291	0.9650	1.2140	0.9460	1.1110
		II	0.9518	1.3884	0.9652	1.5054	0.9419	1.3400
	b	I	0.9510	2.5059	0.9650	2.6879	0.9448	2.4445
		II	0.9540	2.9896	0.9682	3.1835	0.9431	2.8884
		III	0.9491	2.8411	0.9601	3.0821	0.9361	2.6989
*ρ*,***π***,m			Score-Bonferroni		Score-Sidak		Score-Dunnet	
			ECP	MIW	ECP	MIW	ECP	MIW
0.3	a	I	0.9517	0.9456	0.9660	1.0125	0.9486	0.9317
		II	0.9560	1.1183	0.9711	1.2023	0.9503	1.0846
	b	I	0.9583	2.0711	0.9708	2.2193	0.9516	2.0217
		II	0.9618	2.5332	0.9730	2.7063	0.9536	2.4393
		III	0.9692	2.4075	0.9773	2.5826	0.9540	2.2554
0.5	a	I	0.9504	1.0254	0.9642	1.0983	0.9470	1.0101
		II	0.9549	1.2215	0.9694	1.3126	0.9486	1.1840
	b	I	0.9513	2.2410	0.9687	2.3938	0.9460	2.1861
		II	0.9609	2.7047	0.9724	2.8749	0.9528	2.6115
		III	0.9644	2.5568	0.9790	2.7477	0.9508	2.4270
0.7	a	I	0.9518	1.0993	0.9656	1.1778	0.9486	1.0827
		II	0.9569	1.3185	0.9697	1.4178	0.9491	1.2777
	b	I	0.9508	2.3924	0.9655	2.5539	0.9448	2.3407
		II	0.9585	2.8388	0.9706	3.0014	0.9475	2.7512
		III	0.9644	2.7373	0.9787	2.9108	0.9504	2.5607

a: *π* = (0.4,0.4,0.4); b: *π* = (0.25,0.375,0.5).

I: m = (50,50,50); II: m = (30,50,80); III: m = (30,100,500).

**Table 4:** The empirical coverage probability (ECP) and the mean interval width (MIW) of 95% CI for proportion ratio (*g* = 4).

*ρ*,***π***,m			Wald-Bonferroni		Wald-Sidak		Wald-Dunnet	
			ECP	MIW	ECP	MIW	ECP	MIW
0.3	a	I	0.9632	1.0088	0.9417	0.9368	0.9571	0.9875
		II	0.9659	1.1546	0.9476	1.0707	0.9555	1.0992
	b	I	0.9654	2.2043	0.9484	2.0467	0.9571	2.1262
		II	0.9724	2.6947	0.9594	2.5069	0.9623	2.5233
		III	0.9712	2.6441	0.9535	2.4566	0.9576	2.4446
0.5	a	I	0.9639	1.0981	0.9441	1.0189	0.9589	1.0745
		II	0.9648	1.2620	0.9503	1.1691	0.9556	1.2001
	b	I	0.9679	2.4001	0.9486	2.2298	0.9598	2.3169
		II	0.9730	2.8915	0.9583	2.7005	0.9607	2.7212
		III	0.9683	2.8376	0.9557	2.6435	0.9524	2.6327
0.7	a	I	0.9689	1.1838	0.9477	1.0975	0.9623	1.1578
		II	0.9673	1.3687	0.9526	1.2670	0.9588	1.3004
	b	I	0.9702	2.5835	0.9506	2.4002	0.9617	2.4916
		II	0.9713	3.0647	0.9591	2.8643	0.9606	2.8860
		III	0.9693	3.0029	0.9575	2.7802	0.9540	2.7847
*ρ*,***π***,m			Profile-Bonferroni		Profile-Sidak		Profile-Dunnet	
			ECP	MIW	ECP	MIW	ECP	MIW
0.3	a	I	0.9551	1.0328	0.9646	1.0803	0.9496	1.0100
		II	0.9539	1.2366	0.9606	1.2997	0.9387	1.1600
	b	I	0.9575	2.3173	0.9674	2.4291	0.9487	2.2249
		II	0.9657	2.9145	0.9729	3.0552	0.9508	2.7300
		III	0.9631	2.8617	0.9671	2.9897	0.9468	2.6504
0.5	a	I	0.9543	1.1270	0.9641	1.1798	0.9491	1.1015
		II	0.9482	1.3615	0.9600	1.4340	0.9336	1.2834
	b	I	0.9595	2.5194	0.9688	2.6395	0.9498	2.4234
		II	0.9639	3.1285	0.9720	3.2662	0.9514	2.9301
		III	0.9547	3.0884	0.9625	3.2011	0.9383	2.8308
0.7	a	I	0.9569	1.2189	0.9669	1.2769	0.9506	1.1905
		II	0.9488	1.4926	0.9628	1.5742	0.9369	1.4047
	b	I	0.9587	2.7223	0.9682	2.8468	0.9478	2.6213
		II	0.9609	3.2946	0.9671	3.4351	0.9422	3.1173
		III	0.9467	3.2144	0.9538	3.3409	0.9310	3.0552
*ρ*,***π***,m			Score-Bonferroni		Score-Sidak		Score-Dunnet	
			ECP	MIW	ECP	MIW	ECP	MIW
0.3	a	I	0.9568	1.0155	0.9667	1.0604	0.9514	0.9938
		II	0.9610	1.1928	0.9698	1.2490	0.9497	1.1329
	b	I	0.9588	2.2174	0.9692	2.3175	0.9500	2.1387
		II	0.9714	2.7438	0.9788	2.8645	0.9572	2.5690
		III	0.9705	2.6988	0.9758	2.8097	0.9541	2.4823
0.5	a	I	0.9564	1.1033	0.9661	1.1525	0.9506	1.0795
		II	0.9634	1.3038	0.9711	1.3658	0.9515	1.2371
	b	I	0.9608	2.4036	0.9702	2.5079	0.9514	2.3198
		II	0.9712	2.9329	0.9771	3.0464	0.9586	2.7590
		III	0.9686	2.8768	0.9764	2.9656	0.9503	2.6843
0.7	a	I	0.9578	1.1863	0.9687	1.2394	0.9533	1.1603
		II	0.9633	1.4088	0.9715	1.4763	0.9530	1.3373
	b	I	0.9594	2.5762	0.9694	2.6903	0.9498	2.4867
		II	0.9676	3.0907	0.9743	3.2139	0.9518	2.9080
		III	0.9660	3.0436	0.9756	3.1695	0.9492	2.8051

a: *π* = (0.4,0.4,0.4,0.4); b: *π* = (0.25,0.375,0.425,0.5).

I: m = (50,50,50,50); II: m = (30,50,80,100); III: m = (30,50,100,500).

**Table 5:** The empirical coverage probability (ECP) and the mean interval width (MIW) of 95% CI for proportion ratio (*g* = 5).

*ρ*,***π***,m			Wald-Bonferroni		Wald-Sidak		Wald-Dunnet	
			ECP	MIW	ECP	MIW	ECP	MIW
0.3	a	I	0.9632	1.0600	0.9388	0.9777	0.9546	1.0331
		II	0.9663	1.2296	0.9510	1.1370	0.9545	1.1639
	b	I	0.9699	2.4150	0.9532	2.2238	0.9597	2.3024
		II	0.9724	2.9981	0.9546	2.7789	0.9558	2.7808
		III	0.9743	2.9189	0.9628	2.6869	0.9558	2.6206
0.5	a	I	0.9656	1.1536	0.9442	1.0589	0.9588	1.1238
		II	0.9666	1.3493	0.9478	1.2449	0.9543	1.2758
	b	I	0.9727	2.6408	0.9549	2.4382	0.9624	2.5194
		II	0.9738	3.2094	0.9561	2.9882	0.9576	2.9969
		III	0.9723	3.1119	0.9592	2.8945	0.9535	2.8277
0.7	a	I	0.9676	1.2367	0.9409	1.1516	0.9606	1.2043
		II	0.9667	1.4642	0.9445	1.3480	0.9556	1.3821
	b	I	0.9714	2.8503	0.9460	2.6266	0.9603	2.7193
		II	0.9686	3.4006	0.9556	3.1580	0.9553	3.1733
		III	0.974	3.3101	0.9619	3.0778	0.9562	3.0001
*ρ*,***π***,m			Profile-Bonferroni		Profile-Sidak		Profile-Dunnet	
			ECP	MIW	ECP	MIW	ECP	MIW
0.3	a	I	0.9526	1.0882	0.9608	1.1225	0.9448	1.0591
		II	0.9528	1.3236	0.9644	1.3774	0.9402	1.2426
	b	I	0.9618	2.5504	0.9698	2.6284	0.9501	2.4237
		II	0.9634	3.2611	0.9698	3.3636	0.9470	3.0198
		III	0.9768	3.1854	0.9771	3.2860	0.9506	2.8588
0.5	a	I	0.9546	1.1875	0.9647	1.2207	0.9450	1.1550
		II	0.9508	1.4679	0.9599	1.5284	0.9345	1.3743
	b	I	0.9635	2.7837	0.9712	2.8910	0.9517	2.6500
		II	0.9664	3.4799	0.9711	3.5792	0.9484	3.2241
		III	0.9711	3.4271	0.9762	3.5346	0.9467	3.0511
0.7	a	I	0.9530	1.2770	0.9607	1.3352	0.9436	1.2413
		II	0.9520	1.6120	0.9587	1.6767	0.9330	1.5042
	b	I	0.9586	3.0100	0.9620	3.0943	0.9467	2.8648
		II	0.9581	3.6429	0.9645	3.7517	0.9396	3.4234
		III	0.9637	3.5429	0.9712	3.6957	0.9432	3.2936
*ρ*,***π***,m			Score-Bonferroni		Score-Sidak		Score-Dunnet	
			ECP	MIW	ECP	MIW	ECP	MIW
0.3	a	I	0.9542	1.0689	0.9638	1.1010	0.9467	1.0415
		II	0.9608	1.2731	0.9726	1.3199	0.9508	1.2020
	b	I	0.9634	2.4310	0.9707	2.5053	0.9523	2.3167
		II	0.9677	3.0540	0.9721	3.1466	0.9516	2.8265
		III	0.9773	2.9760	0.9807	3.0692	0.9539	2.6676
0.5	a	I	0.9566	1.1612	0.9671	1.1918	0.9485	1.1310
		II	0.9619	1.3988	0.9682	1.4478	0.9488	1.3193
	b	I	0.9643	2.6418	0.9708	2.7265	0.9538	2.5198
		II	0.9694	3.2419	0.9741	3.3346	0.9528	3.0321
		III	0.9746	3.1572	0.9794	3.2316	0.9498	2.8805
0.7	a	I	0.9545	1.2413	0.9616	1.2944	0.9462	1.2087
		II	0.9588	1.5114	0.9652	1.5663	0.9464	1.4246
	b	I	0.9559	2.8322	0.9597	2.9083	0.9455	2.7023
		II	0.9570	3.4054	0.9617	3.5069	0.9413	3.1633
		III	0.9684	3.3396	0.9754	3.4821	0.9467	2.9993

a: *π* = (0.4,0.4,0.4,0.4,0.4); b: *π* = (0.25,0.375,0.425,0.5,0.54).

I: m = (50,50,50,50,50); II: m = (30,45,60,75,90); III: m = (30,50,100,200,500).

An extensive simulation study is conducted for group *g* = 3, 4, 5 with balanced designs having sample size *m*_*i*_ = 20, 40, 80, 500. In this part of the study, 1000 sets of $\vec{\pi }=(\pi_{1},\pi_{2},\cdots ,\pi_{g})$ and *ρ* are randomly generated from the uniform distribution ***U*** (0,1) subject to the condition that each corresponding response probability adhered to the formula (1). The values of *π*_*i*_ are sorted in increasing order, ensuring that the ratios between each *i*^*th*^ group (*i* = 2, ⋯, *g*) and the 1^*st*^ group are consistently greater than 1. This arrangement facilitates a straightforward comparison of the MIW.

For each configuration setting, 10,000 replications are generated, and 95% confidence intervals are constructed. The ECP and MIW are calculated for each method to assess their performance. Additionally, boxplots are created to enable visual comparisons among different methods.

**Fig 1:**
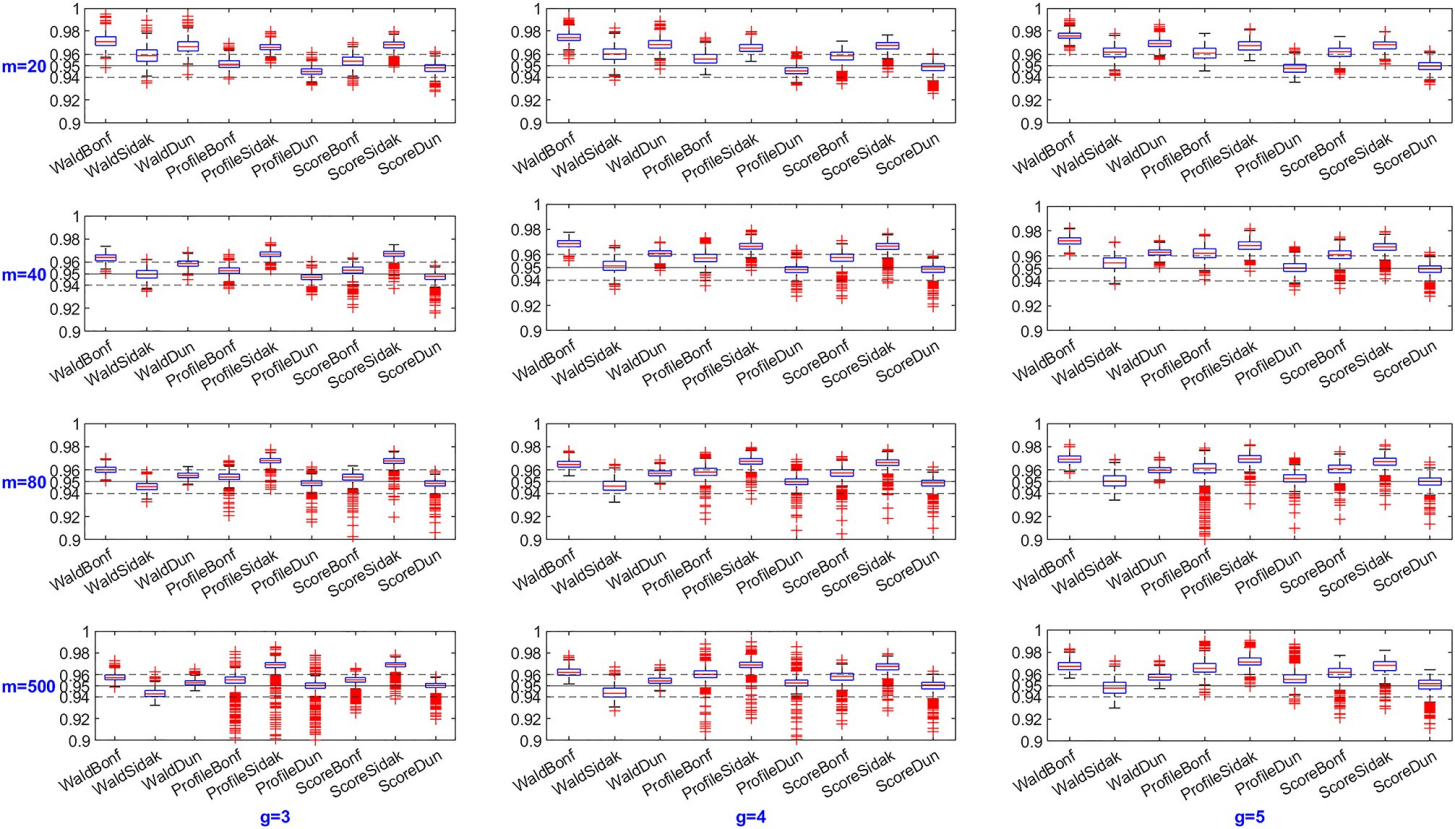
Boxplots of Empirical Coverage Probabilities (ECP).

**Fig 2:**
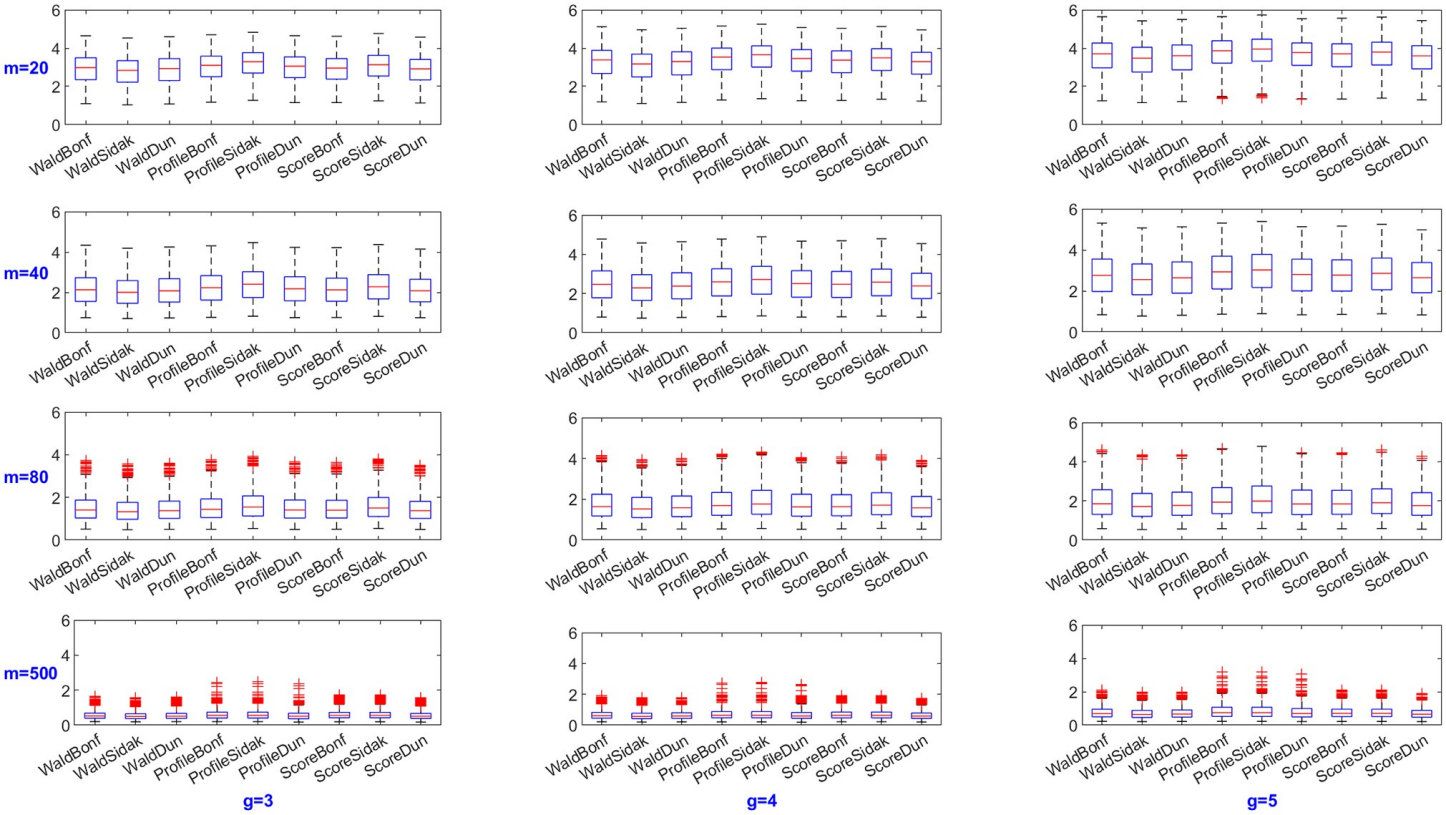
Boxplots of Mean Intercal Width (MIW).

Figs 1 and 2 illustrate the overall distribution of ECP and MIW for all SCIs methods. Among all the proposed methods, the Score method with Dunnett multiplicity adjustment performed best, achieving an average ECP closest to the pre-specified nominal level and the shortest MIW. As shown in Fig 1, as the sample size increases, the ECP of the Profile and Score methods does not show any obvious pattern, while the Wald method approaches the nominal level. The Wald method, when paired with Sidak adjustment method, yields results closer to the pre-specified nominal level (0.95), indicating greater reliability compared to the other two adjustment methods. However, the Sidak correction does not yield an improvement when combined with the profile likelihood and score methods. Meanwhile, Fig 2 demonstrates that the MIW becomes shorter as the sample size increases since asymptotic methods tend to perform better with larger sample sizes. The MIW exhibits a slight increase as the number of group increases, which is an expected trend. This happens because the MIW is calculated as the average of all SCIs. Given that the *π*_*i*_ are sorted in ascending order and the ratios between group (*i* = 2, ⋯, *g*) and the 1^*st*^ group are consistently greater than one and increasing, the addition of more intervals as the number of groups grows leads to a slightly larger MIW.

Additionally, the MIW does not exhibit an obvious difference when comparing the Bonferroni and Dunnett multiplicity adjustments. However, the ECP showes a distinct difference. As expected, the Bonferroni method is more conservative, while the Dunnett method, although computationally more demanding, is justified for its benefits. Overall, score SCI with Dunnett multiplicity adjustment is highly recommeded.

## Real case example

The dataset used for this analysis is sourced from Rosner [2]. It includes information on 218 patients aged 20–39, who were diagnosed with retinitis pigmentosa (RP) and were seen at the Massachusetts Eye and Ear Infirmary from 1970 to 1979. The patients were divided into four groups based on their genetic type: autosomal dominant RP (DOM), autosomal recessive RP (AR), sex-linked RP (SL), and isolated RP (ISO). To simplify the analysis, each patient was associated with a unique family and then randomly selected for the study. The Snellen visual acuity (VA) of an eye was considered affected if it was 20/50 or worse, and normal if it was 20/40 or better. For this analysis, a subgroup of 216 individuals was selected from a total of 218, all of whom had complete VA information for both eyes. Detail information is presented in Table 6.

**Table 6:** Number of affected eyes per person in each group.

Number of affected eyes	Genetic type			
	ISO	DOM	AR	SL
0	67	15	7	3
1	24	6	5	2
2	57	7	9	14

According to Liu and Ma [20] and Tang et al. [21], following a goodness-of-fit test, the equal correlation coefficient model (*ρ* model) is found appropriate for analyzing this dataset. In the *ρ* model, the MLE $\hat{\pi_{i}}$ values are similar to the sample proportion, indicating a strong fit to the model. The estimated values for the parameters are: $\hat{\rho }=0.6416$, ${\hat{\pi }}_{ISO}=0.4658$, ${\hat{\pi }}_{DOM}=0.3625$, ${\hat{\pi }}_{AR}=0.5455$, and ${\hat{\pi }}_{SL}=0.7926$. When calculating the SCI, ISO is considered the control group based on evidence suggesting that RP may occur as an isolated sporadic disorder, without genetic links [22]. The 95% SCI between DOM, AR, SL, and ISO are presented in Table 7.

**Table 7:** 95% SCI of the proportion ratio of affected rates among these groups.

Methods	CIs		
	DOM/ISO	AR/ISO	SL/ISO
WaldBonf	(0.4380, 1.3825)	(0.7302, 1.8782)	(1.2406, 2.3339)
WaldSidak	(0.4552, 1.3304)	(0.7536, 1.8199)	(1.2671, 2.2851)
WaldDun	(0.4389, 1.3798)	(0.7314, 1.8752)	(1.2420, 2.3313)
ProfileBonf	(0.4016, 1.2719)	(0.6696, 1.7442)	(1.1507, 2.2621)
ProfileSidak	(0.3892, 1.2941)	(0.6516, 1.7690)	(1.1281, 2.2870)
ProfileDun	(0.4027, 1.2700)	(0.6711, 1.7421)	(1.1527, 2.2600)
ScoreBonf	(0.4242, 1.2665)	(0.6880, 1.7218)	(1.1257, 2.2434)
ScoreSidak	(0.4134, 1.2882)	(0.6715, 1.7450)	(1.1021, 2.2667)
ScoreDun	(0.4252, 1.2647)	(0.6894, 1.7199)	(1.1277, 2.2414)

Confidence intervals are relatively straightforward to interpret and apply in statistical analysis. According to prior simulation results, the Score method with Dunnett adjustment has been identified as the most effective approach. Additionally, the Wald method with Sidak correction also demonstrates a competitive advantage, particularly in terms of computational efficiency. In simple terms, we examine whether the confidence interval includes the value 1, given that the statistical measure of interest in this context is a ratio. If the confidence interval does not contain 1, it indicates a statistically significant difference. On the other hand, if 1 is included within the confidence interval, it suggests that there is no statistically significant difference. The results show that the CI between DOM and ISO, AR, and ISO contained 1, indicating no significant difference. However, the affected rate in the SL group is significantly greater than in ISO, as the lower bounds of the CIs are greater than 1.

## Discussions

In this study, nine asymptotic SCIs are derived for the ratio of proportions, with these methods being better suited for large sample sizes. To ensure robustness for smaller sample sizes, exact methods are planned for future research. To control the Type I error rate, this study also compared different multiplicity adjustment methods, specifically focusing on the Bonferroni, Sidak and Dunnett methods, with the latter demonstrating better performance. The Bonferroni method has been criticized for its conservative nature, leading to consideration of alternative approaches, such as the Holm method and modified Bonferroni method [23]. However, since these multiplicity adjustment methods are not well-suited to our current computational algorithm, the development of new algorithms may be necessary for future research.

As the group size increases, combining the Dunnett method with an iteration method can become quite time-consuming. However, with advancements in computing technology, a broader range of methods can be explored. Future research could consider a wider range of multiplicity adjustment methods to find the most suitable SCIs.

Furthermore, the asymptotic SCIs proposed in this article are suitable for bilateral data. A potential further research could involve combining unilateral and bilateral data to develop more appropriate SCIs.

## Supporting information

S1 Appendix (PDF)
