# Supplementary material for: Simultaneous confidence interval construction for many-to-one of proportion ratios of bilateral correlated data
Source: PLoS One. 2024 Oct 15;19(10):e0311850. doi: 10.1371/journal.pone.0311850 (PMC11478910; doi:10.1371/journal.pone.0311850)
Supplement: S1 Appendix — (PDF) [file pone.0311850.s001.pdf]

Appendix

Information matrix for computing unconstrained MLE.

Differentiating $l_1(\pi_1, \cdots, \pi_g; \rho)$ with respect to $\pi_i, i = 1, 2, \ldots, g$ and ρ yields

$$\begin{aligned}
\frac{\partial^2 l_1}{\partial \pi_i} &= \frac{-2{\pi_i}^2\rho^2 + 4{\pi_i}^2\rho - 2{\pi_i}^2 + 2\pi_i\rho^2 - 6\pi_i\rho + 4\pi_i - \rho^2 + 2\rho - 2}{(\pi_i - 1)^2(\pi_i\rho - \pi_i + 1)^2} m_{0i} \\
&\quad + \frac{-2{\pi_i}^2 + 2\pi_i - 1}{\pi_i^2(\pi_i - 1)^2} m_{1i} + \frac{-2{\pi_i}^2\rho^2 + 4{\pi_i}^2\rho - 2{\pi_i}^2 + 2\pi_i\rho^2 - 2\pi_i\rho - \rho^2}{{\pi_i}^2(\pi_i + \rho - \pi_i\rho)^2} m_{2i}, \\
\frac{\partial^2 l_1}{\partial \pi_i \partial \rho} &= \frac{1}{(\pi_i\,\rho - \pi_i + 1)^2} m_{0i} - \frac{1}{(\pi_i + \rho - \pi_i\,\rho)^2} m_{2i}, i = 1, 2, \ldots, g, \\
\frac{\partial^2 l_1}{\partial \pi_i \partial \pi_j} &= 0, i \neq j, \quad j = 1, 2, \ldots, g, \\
\frac{\partial^2 l_1}{\partial \rho^2} &= -\sum_{i=1}^{g} [\frac{{\pi_i}^2}{(\pi_i\,\rho - \pi_i + 1)^2} m_{0i} + \frac{1}{(\rho - 1)^2} m_{1i} + \frac{(\pi_i - 1)^2}{(\pi_i - \pi_i\rho + \rho)^2} m_{2i}]
\end{aligned}$$

The Fisher information matrix is given by

$$I\left(\pi_1, \pi_2, \ldots, \pi_g, \rho\right) = \begin{pmatrix} I_{1,1} & I_{1,2} & \cdots & 0 & I_{1,g+1} \\ I_{2,1} & I_{2,2} & \cdots & 0 & I_{2,g+1} \\ \vdots & \vdots & \ddots & \vdots & \vdots \\ I_{g,1} & I_{g,2} & \cdots & I_{g,g} & I_{g,g+1} \\ I_{g+1,1} & I_{g+1,2} & \cdots & I_{g+1,g} & I_{g+1,g+1} \end{pmatrix},$$

$$\begin{aligned}
I_{i,i} &= E(-\frac{\partial^2 l_1}{\partial \pi_i}) = -\frac{m_i\left(-4\,{\pi_i}^2\,\rho^2 + 6\,{\pi_i}^2\,\rho - 2\,{\pi_i}^2 + 4\,\pi_i\,\rho^2 - 6\,\pi_i\,\rho + 2\,\pi_i - \rho^2 + 2\,\rho\right)}{\pi_i\,(\pi_i - 1)\,(-{\pi_i}^2\,\rho^2 + 2\,{\pi_i}^2\,\rho - {\pi_i}^2 + \pi_i\,\rho^2 - 2\,\pi_i\,\rho + \pi_i + \rho)} \\
I_{i,g+1} &= E(-\frac{\partial^2 l_1}{\partial \pi_i \partial \rho}) = \frac{m_i\,\rho\,(2\,\pi_i - 1)}{-{\pi_i}^2\,\rho^2 + 2\,{\pi_i}^2\,\rho - {\pi_i}^2 + \pi_i\,\rho^2 - 2\,\pi_i\,\rho + \pi_i + \rho}, i = 1, 2, \ldots, g \\
I_{i,j} = I_{j,i} &= E(-\frac{\partial^2 l_1}{\partial \pi_i \partial \pi_j}) = 0, i \neq j, j = 1, 2, \ldots, g, \\
I_{g+1,g+1} &= E(-\frac{\partial^2 l_1}{\partial \rho^2}) = \sum_{i=1}^{g} \frac{m_i\,\pi_i\,(\pi_i - 1)\,(\rho + 1)}{(\rho - 1)(\pi_i\,\rho - \pi_i + 1)(\pi_i + \rho - \pi_i\rho)}
\end{aligned}$$

Information matrix for Score statistics.

Differentiating $l_2(\delta_2, \pi_1, \pi_3, \cdots, \pi_g; \rho)$ with respect to $\pi_i, i = 3, \ldots, g$ and ρ yields

$$\begin{aligned}
\frac{\partial^2 l_2}{\partial \delta_2^2} &= \frac{-{\pi_1}^2 \left(2\,{\delta_2}^2\,{\pi_1}^2\,\rho^2 - 4\,{\delta_2}^2\,{\pi_1}^2\,\rho + 2\,{\delta_2}^2\,{\pi_1}^2 - 2\,\delta_2\,\pi_1\,\rho^2 + 6\,\delta_2\,\pi_1\,\rho - 4\,\delta_2\,\pi_1\right)}{\left(\delta_2\,\pi_1 - 1\right)^2 \left(\delta_2\,\pi_1\,\rho - \delta_2\,\pi_1 + 1\right)^2} m_{02} \\
&+ \frac{-{\pi_1}^2 \left(\rho^2 - 2\,\rho + 2\right)}{\left(\delta_2\,\pi_1 - 1\right)^2 \left(\delta_2\,\pi_1\,\rho - \delta_2\,\pi_1 + 1\right)^2} m_{02} \\
&- \frac{2\,{\delta_2}^2\,{\pi_1}^2 - 2\,\delta_2\,\pi_1 + 1}{{\delta_2}^2 \left(\delta_2\,\pi_1 - 1\right)^2} m_{12} \\
&- \frac{2\,{\delta_2}^2\,{\pi_1}^2\,\rho^2 - 4\,{\delta_2}^2\,{\pi_1}^2\,\rho + 2\,{\delta_2}^2\,{\pi_1}^2 - 2\,\delta_2\,\pi_1\,\rho^2 + 2\,\delta_2\,\pi_1\,\rho + \rho^2}{{\delta_2}^2 \left(\rho + \delta_2\,\pi_1 - \delta_2\,\pi_1\,\rho\right)^2} m_{22}, \\
\frac{\partial^2 l_2}{\partial \pi_1 \partial \delta_2} &= \frac{\partial^2 l_2}{\partial \delta \partial \pi_1} = -\frac{{\delta_2}^2\,{\pi_1}^2\,\rho^2 - 3\,{\delta_2}^2\,{\pi_1}^2\,\rho + 2\,{\delta_2}^2\,{\pi_1}^2 + 4\,\delta_2\,\pi_1\,\rho - 4\,\delta_2\,\pi_1 - \rho + 2}{\left(\delta_2\,\pi_1 - 1\right)^2 \left(\delta_2\,\pi_1\,\rho - \delta_2\,\pi_1 + 1\right)^2} m_{02} \\
&\quad - \frac{1}{\left(\delta_2\,\pi_1 - 1\right)^2} m_{12} - \frac{\rho\left(\rho - 1\right)}{\left(\rho + \delta_2\,\pi_1 - \delta_2\,\pi_1\,\rho\right)^2} m_{22}, \\
\frac{\partial^2 l_2}{\partial \pi_i \partial \delta_2} &= 0, \quad i = 3, \ldots, g \\
\frac{\partial^2 l_2}{\partial \rho \partial \delta_2} &= \frac{\partial^2 l_2}{\partial \delta_2 \partial \rho} = \frac{\pi_1}{\left(\delta_2\,\pi_1\,\rho - \delta_2\,\pi_1 + 1\right)^2} m_{02} + \frac{{\pi_1}^4 \left(\delta_2\,\pi_1 - 1\right)\left(\rho + 2\,\delta_2\,\pi_1 - 2\,\delta_2\,\pi_1\,\rho\right)}{\delta_2} m_{22} \\
&\quad - \frac{2\,\delta_2\,\pi_1 - 1}{\delta_2\left(\rho + \delta_2\,\pi_1 - \delta_2\,\pi_1\,\rho\right)} m_{22} - \delta_2\,\pi_1\,\rho\left(\delta_2\,\pi_1 - 1\right)^2 m_{22}, \\
\frac{\partial^2 l_2}{\partial \pi_1^2} &= -\frac{2\,{\pi_1}^2\,\rho^2 - 4\,{\pi_1}^2\,\rho + 2\,{\pi_1}^2 - 2\,\pi_1\,\rho^2 + 6\,\pi_1\,\rho - 4\,\pi_1 + \rho^2 - 2\,\rho + 2}{\left(\pi_1 - 1\right)^2 \left(\pi_1\,\rho - \pi_1 + 1\right)^2} m_{01} \\
&- \frac{2\,{\pi_1}^2 - 2\,\pi_1 + 1}{{\pi_1}^2 \left(\pi_1 - 1\right)^2} m_{11} - \frac{2\,{\pi_1}^2\,\rho^2 - 4\,{\pi_1}^2\,\rho + 2\,{\pi_1}^2 - 2\,\pi_1\,\rho^2 + 2\,\pi_1\,\rho + \rho^2}{{\pi_1}^2 \left(\pi_1 + \rho - \pi_1\,\rho\right)^2} m_{21} \\
&- \frac{{\delta_2}^2 \left(2\,{\delta_2}^2\,{\pi_1}^2\,\rho^2 - 4\,{\delta_2}^2\,{\pi_1}^2\,\rho + 2\,{\delta_2}^2\,{\pi_1}^2 - 2\,\delta_2\,\pi_1\,\rho^2 + 6\,\delta_2\,\pi_1\,\rho - 4\,\delta_2\,\pi_1\right)}{\left(\delta_2\,\pi_1 - 1\right)^2 \left(\delta_2\,\pi_1\,\rho - \delta_2\,\pi_1 + 1\right)^2} m_{02} \\
&- \frac{{\delta_2}^2 \left(\rho^2 - 2\,\rho + 2\right)}{\left(\delta_2\,\pi_1 - 1\right)^2 \left(\delta_2\,\pi_1\,\rho - \delta_2\,\pi_1 + 1\right)^2} m_{02} \\
&- \frac{2\,{\delta_2}^2\,{\pi_1}^2 - 2\,\delta_2\,\pi_1 + 1}{{\pi_1}^2 \left(\delta_2\,\pi_1 - 1\right)^2} m_{12} \\
&- \frac{2\,{\delta_2}^2\,{\pi_1}^2\,\rho^2 - 4\,{\delta_2}^2\,{\pi_1}^2\,\rho + 2\,{\delta_2}^2\,{\pi_1}^2 - 2\,\delta_2\,\pi_1\,\rho^2 + 2\,\delta_2\,\pi_1\,\rho + \rho^2}{{\pi_1}^2 \left(\rho + \delta_2\,\pi_1 - \delta_2\,\pi_1\,\rho\right)^2} m_{22}, \\
\frac{\partial^2 l_2}{\partial \pi_1 \partial \pi_i} &= \frac{\partial^2 l_2}{\partial \pi_i \partial \pi_1} 0, \quad i = 3, \ldots, g
\end{aligned}$$

$$
\begin{aligned}
\frac{\partial^2 l_2}{\partial\rho\partial\pi_1} &= \frac{\partial^2 l_2}{\partial\pi_1\partial\rho} = \frac{1}{\left(\pi_1\,\rho-\pi_1+1\right)^2}m_{01} - \frac{2\pi_1-1}{{\pi_1}^2-\pi_1\,\rho\left(\pi_1-1\right)}m_{21}\\
&- \frac{\left(\pi_1-1\right)\left(\pi_1\,\rho-2\,\pi_1+\rho(\pi_1-1)\right)}{\pi_1}m_{21} - \pi_1\,\rho\left(\pi_1-1\right)^2 m_{21}\\
&+ \frac{\delta_2}{\left(\delta_2\,\pi_1\,\rho-\delta_2\,\pi_1+1\right)^2}m_{02} + {\pi_1}^3(\delta_2\,\pi_1-1)\left(\rho+2\,\delta_2\,\pi_1-2\,\delta_2\,\pi_1\,\rho\right)m_{22}\\
&- \frac{2\,\delta_2\,\pi_1-1}{\pi_1\left(\rho+\delta_2\,\pi_1-\delta_2\,\pi_1\,\rho\right)}m_{22} - \delta_2\,\pi_1\,\rho\left(\delta_2\,\pi_1-1\right)^2 m_{22},\\
\frac{\partial^2 l_2}{\partial\pi_i^2} &= -\frac{2\,{\pi_i}^2\,\rho^2-4\,{\pi_i}^2\,\rho+2\,{\pi_i}^2-2\,\pi_i\,\rho^2+6\,\pi_i\,\rho-4\,\pi_i+\rho^2-2\,\rho+2}{\left(\pi_i-1\right)^2\left(\pi_i\,\rho-\pi_i+1\right)^2}m_{0i}\\
&- \frac{2\,{\pi_i}^2-2\,\pi_i+1}{{\pi_i}^2\left(\pi_i-1\right)^2}m_{1i} - \frac{2\,\rho-2}{{\pi_i}^2-\pi_i\,\rho\left(\pi_i-1\right)}m_{2i}\\
&- \frac{\left(\pi_i\,\rho-2\,\pi_i+\rho\left(\pi_i-1\right)\right)^2}{{\pi_i}^2}m_{2i} - \pi_i\,\rho\left(\pi_i-1\right)^2 m_{2i}, \quad i=3,\ldots,g\\
\frac{\partial^2 l_2}{\partial\pi_i\partial\rho} &= \frac{\partial^2 l_2}{\partial\rho\partial\pi_i} = \frac{1}{\left(\pi_i\,\rho-\pi_i+1\right)^2}m_{0i} - \frac{2\,\pi_i-1}{{\pi_i}^2-\pi_i\,\rho\left(\pi_i-1\right)}m_{2i}\\
&- \frac{\left(\pi_i-1\right)\left(\pi_i\,\rho-2\,\pi_i+\rho\left(\pi_i-1\right)\right)}{\pi_i}m_{2i} - \pi_i\,\rho\left(\pi_i-1\right)^2 m_{2i}, \quad i=3,\ldots,g\\
\frac{\partial^2 l_2}{\partial\rho^2} &= -\frac{m_{01}{\pi_1}^2}{\left(\pi_1\,\rho-\pi_1+1\right)^2} - \frac{m_{11}}{\left(\rho-1\right)^2} - \frac{m_{21}\,{\pi_1}^2\left(\pi_1-1\right)^2}{\left({\pi_1}^2-\pi_1\,\rho\left(\pi_1-1\right)\right)^2}\\
&- \frac{{\delta_2}^2\,m_{02}\,{\pi_1}^2}{\left(\delta_2\,\pi_1\,\rho-\delta_2\,\pi_1+1\right)^2} - \frac{m_{12}}{\left(\rho-1\right)^2} - \frac{{\delta_2}^2\,m_{22}\,{\pi_1}^2\left(\delta_2\,\pi_1-1\right)^2}{\left({\delta_2}^2\,{\pi_1}^2-\delta_2\,\pi_1\,\rho\left(\delta_2\,\pi_1-1\right)\right)^2}\\
&- \sum_{i=3}^{g}\Big[\frac{m_{0\mathrm{i}}\,{\pi_i}^2}{\left(\pi_i\,\rho-\pi_i+1\right)^2} + \frac{m_{1\mathrm{i}}}{\left(\rho-1\right)^2} + \frac{m_{2\mathrm{i}}\,{\pi_i}^2\left(\pi_i-1\right)^2}{\left({\pi_i}^2-\pi_i\,\rho\left(\pi_i-1\right)\right)^2}\Big],
\end{aligned}
$$

The Fisher information matrix is given by

$$
I\left(\delta_2,\pi_1,\pi_i,\ldots,\pi_g,\rho\right) = \begin{pmatrix} I_{1,1} & I_{1,2} & 0 & \cdots & I_{1,g+1}\\ I_{1,2} & I_{2,2} & 0 & \cdots & I_{2,g+1}\\ 0 & 0 & I_{i,i} & \cdots & I_{i,g+1}\\ \vdots & \vdots & \vdots & \ddots & \vdots\\ 0 & 0 & I_{i,g+1} & \cdots & I_{g+1,g+1} \end{pmatrix},
$$

$$
\begin{aligned}
I_{1,1} = E(-\frac{\partial^2 l_2}{\partial \delta_2^2}) &= -2\, m_2\, {\pi_1}^2\, (\rho-1) + \frac{m_2\, {\pi_1}^2\, (\rho + 2\, \delta_2\, \pi_1 - 2\, \delta_2\, \pi_1\, \rho - 2)}{\delta_2\, \pi_1 - 1} \\
&+ \frac{2\, m_2\, \pi_1\, (2\, \delta_2\, \pi_1 - 1)\, (\rho-1)}{\delta_2} + \frac{m_2\, {\pi_1}^2\, (\rho-1)\, (\rho + 2\, \delta_2\, \pi_1 - 2\, \delta_2\, \pi_1\, \rho - 2)}{\delta_2\, \pi_1\, \rho - \delta_2\, \pi_1 + 1} \\
&+ \frac{m_2\, (\delta_2\, {\pi_1}^2 - \delta_2\, \pi_1\, \rho\, (\delta_2\, \pi_1 - 1))\, {(\rho + 2\, \delta_2\, \pi_1 - 2\, \delta_2\, \pi_1\, \rho)}^2}{{\delta_2}^2\, {(\rho + \delta_2\, \pi_1 - \delta_2\, \pi_1\, \rho)}^2} \\
&+ \frac{2\, m_2\, {\pi_1}^2\, (2\, \delta_2\, \pi_1 - 1)\, (\rho-1)}{\delta_2\, \pi_1 - 1} + \frac{2\, m_2\, \pi_1\, (\delta_2\, {\pi_1}^2 - \delta_2\, \pi_1\, \rho\, (\delta_2\, \pi_1 - 1))\, (\rho-1)}{\delta_2\, (\rho + \delta_2\, \pi_1 - \delta_2\, \pi_1\, \rho)},
\end{aligned}
$$

$$
\begin{aligned}
I_{1,2} = E(-\frac{\partial^2 l_2}{\partial \pi_1 \partial \delta_2}) &= -m_2\, (\rho + 4\, \delta_2\, \pi_1 - 4\, \delta_2\, \pi_1\, \rho - 2) + 2\, m_2\, (2\, \delta_2\, \pi_1 - 1)\, (\rho-1) \\
&- 2\, m_2\, (4\, \delta_2\, \pi_1 - 1)\, (\rho-1) + \frac{\delta_2\, m_2\, \pi_1\, (\rho + 2\, \delta_2\, \pi_1 - 2\, \delta_2\, \pi_1\, \rho - 2)}{\delta_2\, \pi_1 - 1} \\
&- \frac{m_2\, (\delta_2\, {\pi_1}^2 - \delta_2\, \pi_1\, \rho\, (\delta_2\, \pi_1 - 1))\, (\rho + 4\, \delta_2\, \pi_1 - 4\, \delta_2\, \pi_1\, \rho)}{\delta_2\, \pi_1\, (\rho + \delta_2\, \pi_1 - \delta_2\, \pi_1\, \rho)} \\
&+ \frac{m_2\, (\delta_2\, {\pi_1}^2 - \delta_2\, \pi_1\, \rho\, (\delta_2\, \pi_1 - 1))\, {(\rho + 2\, \delta_2\, \pi_1 - 2\, \delta_2\, \pi_1\, \rho)}^2}{\delta_2\, \pi_1\, {(\rho + \delta_2\, \pi_1 - \delta_2\, \pi_1\, \rho)}^2} \\
&+ \frac{\delta_2\, m_2\, \pi_1\, (\rho-1)\, (\rho + 2\, \delta_2\, \pi_1 - 2\, \delta_2\, \pi_1\, \rho - 2)}{\delta_2\, \pi_1\, \rho - \delta_2\, \pi_1 + 1} + \frac{2\, \delta_2\, m_2\, \pi_1\, (2\, \delta_2\, \pi_1 - 1)\, (\rho-1)}{\delta_2\, \pi_1 - 1},
\end{aligned}
$$

$$
I_{1,i} = E(-\frac{\partial^2 l_2}{\partial \pi_i \partial \delta_2}) = 0, \quad i = 3, \ldots, g
$$

$$
\begin{aligned}
I_{1,g+1} = E(-\frac{\partial^2 l_2}{\partial \rho \partial \delta_2}) &= \frac{m_2\, \pi_1\, \left(-{\delta_2}^3\, {\pi_1}^3\, \rho + {\delta_2}^3\, {\pi_1}^3 + {\delta_2}^2\, {\pi_1}^3\, \rho - {\delta_2}^2\, {\pi_1}^3 + 2\, {\delta_2}^2\, {\pi_1}^2\, \rho^2\right)}{(\delta_2\, \pi_1\, \rho - \delta_2\, \pi_1 + 1)\, {(\rho + \delta_2\, \pi_1 - \delta_2\, \pi_1\, \rho)}^2} \\
&\frac{m_2\, \pi_1\, \left(2\, {\delta_2}^2\, {\pi_1}^2\, \rho - {\delta_2}^2\, {\pi_1}^2 + \delta_2\, {\pi_1}^2 + 3\, \delta_2\, \pi_1\, \rho^2 - \delta_2\, \pi_1\, \rho - \rho^2\right)}{(\delta_2\, \pi_1\, \rho - \delta_2\, \pi_1 + 1)\, {(\rho + \delta_2\, \pi_1 - \delta_2\, \pi_1\, \rho)}^2},
\end{aligned}
$$

$$
\begin{aligned}
I_{2,2} = E(-\frac{\partial^2 l_2}{\partial \pi_1^2}) &= (-3\, \rho + \frac{(2\, \pi_1 + \rho - 2\, \pi_1\, \rho - 2)}{\pi_1 - 1} + \frac{{(2\, \pi_1 + \rho - 2\, \pi_1\, \rho)}^2}{\pi_1} + \pi_1\, \rho^2) m_1 \\
&+ (\frac{(4\, \pi_1 - 2)\, (\rho-1)}{\pi_1} + \frac{(4\, \pi_1 - 2)\, (\rho-1)}{\pi_1 - 1} + \frac{\pi_1\, (2\, \rho - 2)}{\pi_1 + \rho - \pi_1\, \rho}) m_1 \\
&+ (\frac{(\rho-1)(2\, \pi_1 + \rho - 2\, \pi_1\, \rho - 2)}{\pi_1\, \rho - \pi_1 + 1} + 2) m_1 - 2\, {\delta_2}^2\, (\rho-1) m_2 \\
&+ (\frac{{\delta_2}^2\, (\rho + 2\, \delta_2\, \pi_1 - 2\, \delta_2\, \pi_1\, \rho - 2)}{\delta_2\, \pi_1 - 1} + \frac{\delta_2\, {(\rho + 2\, \delta_2\, \pi_1 - 2\, \delta_2\, \pi_1\, \rho)}^2}{{(\rho + \delta_2\, \pi_1 - \delta_2\, \pi_1\, \rho)}^2}) m_2 \\
&+ \frac{{\delta_2}^2\, (\rho-1)\, (\rho + 2\, \delta_2\, \pi_1 - 2\, \delta_2\, \pi_1\, \rho - 2)}{\delta_2\, \pi_1\, \rho - \delta_2\, \pi_1 + 1} m_2 \\
&+ (\frac{2\, {\delta_2}^2\, \pi_1\, (\rho-1)}{(\rho + \delta_2\, \pi_1 - \delta_2\, \pi_1\, \rho)} + \frac{2\, {\delta_2}^2\, (2\, \delta_2\, \pi_1 - 1)\, (\rho-1)}{\delta_2\, \pi_1 - 1} + \frac{2\, \delta_2\, (2\, \delta_2\, \pi_1 - 1)\, (\rho-1)}{\pi_1}) m_2,
\end{aligned}
$$

$$I_{2,i} = E(-\frac{\partial^2 l_2}{\partial \pi_1 \partial \pi_i}) = 0, \quad i = 3, \ldots, g$$

$$I_{2,g+1} = E(-\frac{\partial^2 l_2}{\partial \rho \partial \pi_1}) = (2\,\pi_1 + \frac{\pi_1\,(2\,\pi_1 + \rho - 2\,\pi_1\,\rho - 2)}{\pi_1\,\rho - \pi_1 + 1} + \frac{\pi_1\,(2\,\pi_1 - 1)}{(\pi_1 + \rho - \pi_1\,\rho)})m_1$$
$$- (\frac{\pi_1\,(\pi_1 - 1)\,(2\,\pi_1 + \rho - 2\,\pi_1\,\rho)}{(\pi_1 + \rho - \pi_1\,\rho)^2} + 1)m_1 + (\delta_2\,(2\,\delta_2\,\pi_1 - 1)\frac{\delta_2\,\pi_1\,(2\,\delta_2\,\pi_1 - 1)}{(\rho + \delta_2\,\pi_1 - \delta_2\,\pi_1\,\rho)})m_2$$
$$+ (\frac{{\delta_2}^2\,\pi_1\,(\rho + 2\,\delta_2\,\pi_1 - 2\,\delta_2\,\pi_1\,\rho - 2)}{\delta_2\,\pi_1\,\rho - \delta_2\,\pi_1 + 1} - \frac{\delta_2\,\pi_1\,(\delta_2\,\pi_1 - 1)\,(\rho + 2\,\delta_2\,\pi_1 - 2\,\delta_2\,\pi_1\,\rho)}{(\rho + \delta_2\,\pi_1 - \delta_2\,\pi_1\,\rho)^2})m_2,$$

$$I_{i,i} = E(-\frac{\partial^2 l_2}{\partial \pi_i^2}) = \frac{4\,{\pi_i}^2\,\rho^2 - 6\,{\pi_i}^2\,\rho + 2\,{\pi_i}^2 - 4\,\pi_i\,\rho^2 + 6\,\pi_i\,\rho - 2\,\pi_i + \rho^2 - 2\,\rho}{\pi_i\,(\pi_i - 1)\,(-{\pi_i}^2\,\rho^2 + 2\,{\pi_i}^2\,\rho - {\pi_i}^2 + \pi_i\,\rho^2 - 2\,\pi_i\,\rho + \pi_i + \rho)},$$

$$I_{i,g+1} = E(-\frac{\partial^2 l_2}{\partial \pi_i \partial \rho}) = \frac{\rho\,(2\,\pi_i - 1)}{-{\pi_i}^2\,\rho^2 + 2\,{\pi_i}^2\,\rho - {\pi_i}^2 + \pi_i\,\rho^2 - 2\,\pi_i\,\rho + \pi_i + \rho},$$

$$I_{g+1,g+1} = E(-\frac{\partial^2 l_2}{\partial \rho^2}) = -\frac{m_1\,{\pi_1}^2\,(\pi_1 - 1)}{\pi_1\,\rho - \pi_1 + 1} + \frac{{\pi_1}^2\left(m_1\,{\pi_1}^2 - \pi_1\,\rho\,(\pi_1 - 1)\right)(\pi_1 - 1)^2}{\left({\pi_1}^2 - \pi_1\,\rho\,(\pi_1 - 1)\right)^2}$$
$$+ \frac{2\,m_1\,\pi_1\,(\pi_1 - 1)}{\rho - 1} + \frac{2\,m_i\,\pi_i\,(\pi_i - 1)}{\rho - 1} + \frac{2\,\delta_2\,m_2\,\pi_1\,(\delta_2\,\pi_1 - 1)}{\rho - 1}$$
$$+ \frac{{\delta_2}^2\,{\pi_1}^2\,(\delta_2\,\pi_1 - 1)^2\left(\delta_2\,m_2\,{\pi_1}^2 - (\delta_2\,\pi_1\,\rho\,(\delta_2\,\pi_1 - 1))\right)}{\left({\delta_2}^2\,{\pi_1}^2 - (\delta_2\,\pi_1\,\rho\,(\delta_2\,\pi_1 - 1))\right)^2} - \frac{{\delta_2}^2\,m_2\,{\pi_1}^2\,(\delta_2\,\pi_1 - 1)}{\delta_2\,\pi_1\,\rho - \delta_2\,\pi_1 + 1}$$
$$- \sum_{i=3}^{g}[\frac{m_i\,{\pi_i}^2\,(\pi_i - 1)}{\pi_i\,\rho - \pi_i + 1} - \frac{\pi_i\,(m_i\,\pi_i - \rho\,(\pi_i - 1))\,(\pi_i - 1)^2}{(\pi_i - \rho\,(\pi_i - 1))^2}],$$
